# Piezoelectric Stimulation Induces Osteogenesis in Mesenchymal Stem Cells Cultured on Electroactive Two-Dimensional Substrates

**DOI:** 10.1021/acsapm.4c02485

**Published:** 2024-11-06

**Authors:** Maria Guillot-Ferriols, Carlos M. Costa, Daniela M. Correia, José Carlos Rodríguez-Hernández, Penelope M. Tsimbouri, Senentxu Lanceros-Méndez, Matthew J. Dalby, José Luis Gómez Ribelles, Gloria Gallego-Ferrer

**Affiliations:** 1 Center for Biomaterials and Tissue Engineering (CBIT), Universitat Politècnica de València, Valencia 46022, Spain; 2 Biomedical Research Networking Center on Bioengineering, Biomaterials and Nanomedicine (CIBER-BBN), Valencia 46022, Spain; 3 Physics Centre of Minho and Porto Universities (CF-UM-UP) and Laboratory of Physics for Materials and Emergent Technologies, LapMET, University of Minho, Braga 4710-057, Portugal; 4 Institute of Science and Innovation for Bio-Sustainability (IB-S), University of Minho, Braga 4710-057, Portugal; 5 Center of Chemistry, Universidade Do Minho, Braga 4710-058, Portugal; 6 Center for the Cellular Microenvironment, School of Molecular Biosciences, College of Medical, Veterinary and Life Sciences, University of Glasgow, Glasgow G12 8QQ, United Kingdom; 7 BCMaterials, Basque Center for Materials, Applications and Nanostructures, UPV/EHU, Science Park, Leioa 48940, Spain; 8 Basque Foundation for Science, IKERBASQUE, Bilbao 48009, Spain

**Keywords:** mesenchymal stem cells, poly(vinylidene) fluoride, ionic liquid, electromechanical stimulation, osteogenesis

## Abstract

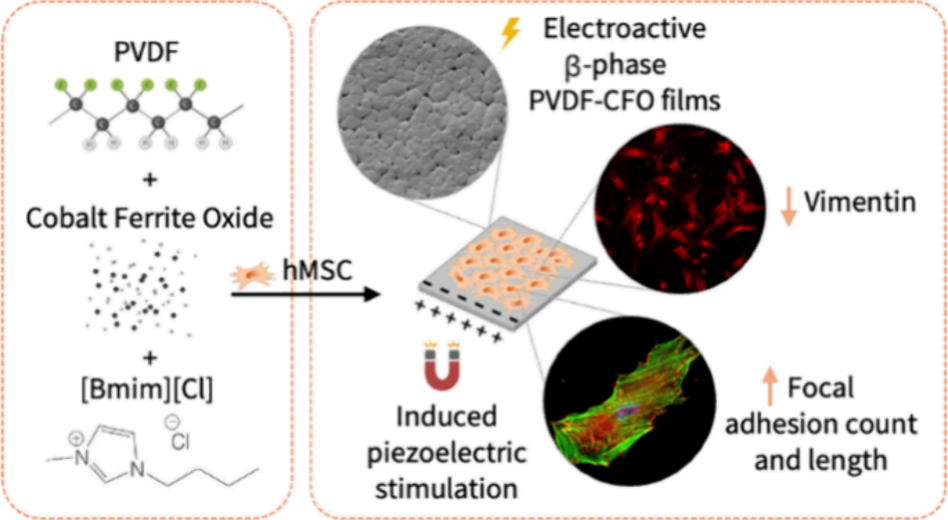

Physical cues have been shown to be effective in inducing osteogenic differentiation of mesenchymal stem cells (MSCs). Here, we propose piezoelectric stimulation as a potential osteogenic cue mimicking the electroactive properties of bone’s extracellular matrix. When combined with a magnetostrictive component, piezoelectric polymers can be used for MSC stimulation by applying an external magnetic field. The deformation of the magnetostrictive component produces a deformation in the polymer matrix, generating a change in the surface charge that induces an electric field that can be transmitted to the cells. Cell adhesion, cytoskeleton changes, and metabolomics are the first evidence of MSC osteoblastogenesis and can be used to study initial MSC response to this kind of stimulation. In the current study, poly(vinylidene) fluoride (PVDF) piezoelectric films with and without cobalt ferrite oxide (CFO) crystallized from the melt in the presence of the ionic liquid 1-butyl-3-methyl-imidazolium chloride ([Bmim][Cl]) were produced. [Bmim][Cl] allowed the production of the β-phase, the most electroactive phase, even without CFO. After ionic liquid removal, PVDF and PVDF-CFO films presented high percentages of the β-phase and similar crystalline content. Incorporating CFO nanoparticles was effective, allowing the electromechanical stimulation of MSCs by applying a magnetic field with a bioreactor. Before stimulation, the initial response of MSCs was characterized in static conditions, showing that the produced films were biocompatible and noncytotoxic, allowing MSC adhesion and proliferation in the short term. Stimulation experiments revealed that MSCs electromechanically stimulated for 3 days in PVDF-CFO supports showed longer focal adhesions and decreased vimentin cytoskeletal density, both signals of early osteogenic differentiation. Furthermore, they rearranged their energy metabolism toward an osteogenic phenotype after 7 days of culture under the same stimulation. The results prove that MSCs respond to electromechanical stimulation by osteogenic differentiation.

## 1 Introduction

MSCs are multipotent cells that can differentiate into osteoblasts, adipocytes, chondrocytes, and reticular cells.^1^ As an autologous stem cell source, they are potential candidates for advanced regeneration therapies, especially for treating bone defects. MSCs are actively implicated in *in vivo* bone repair. They migrate to the injured site and differentiate into osteoblasts in response to biochemical and biophysical stimuli in the bone’s microenvironment.^2^

Many proposed therapies using MSCs rely on their potential to differentiate once they are injected into the injured site. Nevertheless, related to some pathologies, the altered homeostasis of the stem cell niche at the bone defect may not provide the right cues to initiate the osteogenic differentiation cascade in MSCs.^3^ For that reason, predifferentiation approaches are usually preferred since the induction of an osteoblastic phenotype before transplantation has shown enhanced healing capacity.^4−7^ Physical cues have been proposed as an alternative to biochemical induction due to their specific potential,^8^ avoiding undesired side effects that may be observed when using media formulations containing dexamethasone.^9^ More precisely, piezoelectric stimulation is gaining research interest since MSCs reside within a collagen matrix that provides an electroactive environment.^10^

PVDF has been explored as a suitable piezoelectric polymer in the design of cell culture supports for the stimulation of osteogenic progenitors.^11−15^ Its combination with a magnetostrictive phase, generating a magnetoelectric material, allows piezoelectric stimulation by applying a magnetic field, a minimally invasive wireless approach. When the magnetic field is applied to the composite, a deformation is induced in the magnetostrictive component. This is transmitted to the piezoelectric matrix, which changes electrical polarization.^16^

This approach inevitably links electrical and mechanical stimulation, which can trigger a mechanotransduction response able to activate intracellular signaling cascades in MSCs, influencing proliferation, migration, and differentiation.^17^ Focal adhesions (FA), multiprotein complexes under the cell membrane, are responsible for mechanosensing, perceiving, and transferring the mechanical cues in the extracellular milieu to the cellular cytoskeleton. They serve as an interface between the integrins, directly contacting the extracellular environment and the actin cytoskeleton.^18^ Changes in the focal adhesion number, length, and cytoskeleton tension directly relate to osteogenesis. Adhesion maturation leads to high intracellular tension and increased tensile forces being applied to the nucleus, affecting gene expression primarily through the mitogen-activated protein kinase (MAPK) pathway. Indeed, extracellular signal-related kinase (ERK1/2), a part of the MAPK family, is known to be activated by the formation of large, mature adhesions leading to the phosphorylation (activation) of runt-related transcription factor 2 (RUNX2), the master transcription factor of the osteogenic differentiation pathway.^19,20^

The biomedical application of PVDF mainly relies on its crystallization in the β-phase, which shows the highest piezoelectric response. This phase is usually obtained through the stretching of α-phase films obtained from the melt, by techniques such as solvent casting at temperatures lower than 70 °C or through the induction of specific fillers such as clays, magnetostrictive nanoparticles or ionic liquids (ILs).^21^

ILs are exclusively composed of ions and with a melting temperature below 100 °C. Their excellent physical–chemical characteristics, such as negligible vapor pressure, high ionic conductivity, and excellent solubility and miscibility with many compounds, have motivated their use as a replacement for organic solvents.^22^ These last two properties could also be of great interest for their use as PVDF nucleating agents, a less explored application. The β-phase polymorph can be induced by the presence of ILs,^23,24^ which, after crystallization, can be easily removed, obtaining a PVDF cell culture support in its most electroactive phase. Specifically, IL removal allows for producing PVDF supports without magnetostrictive nanoparticles with the same structure, electroactive, and crystalline composition as the ones containing them, which can be used as controls for stimulation experiments.

Taking all of this into account, we developed PVDF films crystallized in the presence of the IL [Bmim][Cl], with or without magnetostrictive nanoparticles, to characterize MSCs response to electromechanical stimulation. Films were characterized before and after IL removal, and their absence of cytotoxicity for MSCs was also evaluated. MSCs’ response to electromechanical stimulation regarding focal adhesion formation and cytoskeleton reorganization was studied by applying a magnetic field using a bioreactor. Untargeted metabolomic analysis was employed to survey cell metabolic changes related to the MSC response to electromechanical stimulation. As far as the authors know, this is the first time that PVDF films crystallized in the presence of the IL [Bmim][Cl] have been used to stimulate MSCs electromechanically.

## 2 Materials and Methods

### 2.1 Electroactive Film Production

PVDF (Solef 6010, Mw ∼ 300 kg·mol^–1^) and PVDF films containing 20% (w/w) of cobalt ferrite oxide spherical nanoparticles (CoFe_2_O_4_, CFO, 35–55 nm size range, Nanostructured & Amorphous Materials) were produced. Both types of films contained a 20% (w/w) content of the IL [Bmim][Cl] (Inc. and Iolitec). The adequate amounts of [Bmim][Cl] and CFO, when applicable, were mixed in 6 mL of N,N-dimethylformamide (DMF, anhydrous, 99.8%, Merck) and ultrasonicated for 3 h in an ultrasound bath. Afterward, the dispersed filler solutions were mixed with 1 g of PVDF powder and mechanically stirred for 3 h to obtain a homogeneous solution. After mixing and dissolving the PVDF polymer, the films were prepared using the doctor blade coating technique onto a glass substrate and placed in an oven (P-Selecta) at 210 °C for 10 min for solvent evaporation.^25^ Films with an average thickness of 60 μm were obtained.

### 2.2 Ionic Liquid Removal and Film Poling Process

Films were placed in ultrapure water for 5 days for IL removal, where every day the water was replaced and the sample weight was measured. After IL removal, the membranes were polarized using the corona method. The polarization process was performed by applying an electric field of ∼10 kV at a constant current of 10 μA for 1 h at a temperature of 120 °C. A broad range d_33_ meter (Model 8000, APC Int Ltd.) was used to measure the d_33_ piezoelectric response with a |5| pC/N value, suitable for biomedical applications and similar to others reported in the literature.^26^ The piezoelectric response is determined by the polymer phase, but the specific macroscopic response is also dependent on polymer microstructure, crystallinity and poling state, among others.^27^

### 2.3 Film Characterization

#### 2.3.1 Field Emission Scanning Electron Microscopy

PVDF and PVDF-CFO film surfaces before and after IL removal were characterized by means of field emission scanning electron microscopy (FESEM) (AURIGA compact, Zeiss). Before washing, films were imaged with an accelerated voltage of 1 kV, and washed films were imaged with a 2 kV voltage. Samples were coated with platinum following a standard sputtering protocol for 90 s (JFC 1100, JEOL).

#### 2.3.2 Fourier Transform Infrared Spectroscopy

Fourier transform infrared (FTIR) spectra were recorded by using an ALPHA FTIR spectrometer (Bruker) in attenuated total reflection (ATR) mode from 4000 to 400 cm^–1^ at a wavelength resolution of 4 cm^–1^. FTIR spectra were obtained after 64 scans for each sample. PVDF and PVDF-CFO samples before and after washing were assayed. Three different samples produced in three different batches were analyzed.

#### 2.3.3 Differential Scanning Calorimetry

Differential scanning calorimetry (DSC) was carried out with a DSC 8000 (PerkinElmer) instrument for scans in the melting region under a flowing nitrogen atmosphere. A sample mass of 2–4 mg was encapsulated in aluminum pans, and thermograms were recorded between 0 and 200 °C at a heating rate of 20 °C/min. Three replicates of PVDF and PVDF-CFO films after washing, produced in three different batches, were used for the measurements.

#### 2.3.4 Vibrating Sample Magnetometer

After IL removal, films containing magnetostrictive nanoparticles were magnetically characterized using a Microsense 2 T vibrating sample magnetometer (VSM). Three different samples produced in three different batches were measured.

Magnetization curves M(H) were evaluated up to 18 kOe, and the actual content of CFO was calculated by comparing the pure CFO saturation magnetization value (60 emu/g) to the one obtained in the composite samples by means of eq 1(28)
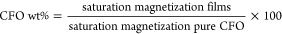
1

### 2.4 Cell Response

Human bone marrow mesenchymal stem cells (PromoCell) were used for the cell culture assays. MSCs were expanded in basal medium containing Dulbecco’s modified Eagle medium (DMEM) high glucose (4.5 g/L) (Gibco) supplemented with 10% (v/v) fetal bovine serum (FBS, Gibco), 4 mM l-glutamine (Lonza), 1× nonessential amino acids (NEAA, Gibco), 1 mM sodium pyruvate (Gibco), 70 U/mL penicillin, 70 μg/mL streptomycin (P/S, Life Technologies), and 0.25 μg/mL fungizone (Life Technologies), at 37 °C in a humidified atmosphere with 5% CO_2_. All experiments were performed at passages not superior to 5.^29,30^

Films were sterilized by performing three washes with ethanol 70% (v/v) under shaking for 10 min each. After, samples were rewashed three times with Dulbecco’s phosphate buffered saline (DPBS, Sigma-Aldrich) and left to dry. 8 mm diameter disks were obtained, and UV was applied for 30 min on each side. 8 mm glass slides were used as noncharged controls in all experiments and were sterilized by UV light for 30 min on each side. Finally, samples were placed in a 48-well plate and silicon rings were used to prevent them from floating. Due to PVDF hydrophobicity, all samples were coated with fibronectin from human plasma (Sigma-Aldrich) on the negatively charged side before cell seeding.^31^ Samples were incubated in a 20 μg/mL fibronectin solution in DPBS for 1 h at room temperature. Afterward, samples were washed twice in DPBS to remove nonadsorbed fibronectin and kept in DPBS until cell seeding.

#### 2.4.1 Initial Mesenchymal Stem Cell Response at Static Mode

##### 2.4.1.1 Cytotoxicity/Leachable Test

Cytotoxicity of CFO nanoparticles and any remaining traces of IL after washing was ruled out by performing a leachable test based on the ISO 10993–5 standard. PVDF and PVDF-CFO films, previously sterilized and placed on a 48-well plate, were incubated with 300 μL of basal medium per well for 24 h at 37 °C in a humidified atmosphere with 5% CO_2_. Latex disks of 8 mm were employed as a positive control, while the basal medium was used as a negative control. Three replicates per condition were used. The leachable solution was analyzed for toxicity using the tetrazolium salt MTS (3-(4,5-dimethylthiazol-2-yl)-5-(3-carboxymethoxyphenyl)-2-(4-sulfophenyl)-2*H*-tetrazolium) assay. This method allows indirect cell viability measurement by determining cells’ mitochondrial activity. MSCs were seeded at a density of 10^4^ cells/cm^2^ in a 48-tissue culture plate and kept in the culture for 24 h. After, the culture medium was replaced with the extraction medium, which was in contact with the materials. Cells were cultured for 24 h. Subsequently, the medium was replaced for DMEM without phenol red (Sigma-Aldrich) containing the MTS reagent (Biovision) at a working dilution of 1:10. Cells were incubated for 2 h at 37 °C. After that, the supernatant was transferred to a new plate, and the optical density at 490 nm was measured on a Victor3 microplate reader (PerkinElmer). For each biological replicate, two technical replicates were analyzed. Cell viability was determined by applying eq 2(15)
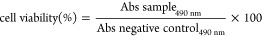
2

##### 2.4.1.2 Cell Spreading

Cell spreading and distribution were assessed by staining F-actin and nuclei. MSCs were seeded on PVDF, PVDF-CFO and glass slide surfaces, previously sterilized and coated with fibronectin, at a density of 5 × 10^3^ cells/cm^2^. Three replicates per condition were used for the assay. Cells were seeded in basal medium without FBS to promote cell adhesion to the fibronectin on the surfaces. A 100 μL drop containing the desired number of cells was deposited inside the silicon ring. After 3 h, the required basal medium and FBS volume for a final concentration of 10% (v/v) were added to each well. This seeding method was used in all the subsequent cell culture experiments.

Cells were cultured for 24 h and then were fixed in 4% (v/v) paraformaldehyde solution (Panreac) for 20 min. Samples were washed three times in DPBS and permeabilized with permeabilization buffer (sucrose 300 mM, NaCl 50 mM, MgCl_2_ hexahydrate 3 mM, HEPES 20 mM, Triton X-100 0.5% (v/v), pH 7.2) for 5 min at 4 °C. Subsequently, they were blocked in 1% (w/v) bovine serum albumin (BSA, Sigma-Aldrich) solution in DPBS/0.1% (v/v) Tween-20 (Sigma-Aldrich) for 1 h at room temperature and incubated with Actin Red 555 ReadyProbes reagent (Fisher Scientific) following manufacturer’s instructions. Then, samples were washed 3 times with DPBS/0.1% (v/v) Tween-20 and mounted with Fluoroshield mounting medium with DAPI (Abcam). Images from four different areas in each one of the three replicates were taken with a fluorescence microscope (Nikon Eclipse 80i), and cell spreading was analyzed using CellProfiler image analysis software (Broad Institute, USA). Briefly, masks of images were obtained from previous segmentation, and cell areas were measured for the scaled images. 85 cells per condition, at least, from the three different replicates were used for the analysis.

##### 2.4.1.3 Proliferation

To determine MSC proliferation, cells were cultured on the supports for 1, 3, and 7 days. 12 h before cell seeding, cells were starved in basal media containing 1% (v/v) FBS to synchronize the cell cycle. PVDF, PVDF-CFO, and glass slides were then seeded at a density of 5 × 10^3^ cells/cm^2^ in basal medium without FBS following the protocol described in Section 2.4.1.2, using three replicates per condition. Cell proliferation was assessed by an MTS assay (protocol in section 2.4.1.1). Cell number was calculated using a calibration curve.

#### 2.4.2 Mesenchymal Stem Cell Response at Dynamic Mode

To electromechanically stimulate MSCs, samples were stimulated using a homemade magnetic bioreactor to generate an alternating magnetic field (0–230 Oe) due to the movement of neodymium magnets below the 48-well tissue culture plate.^32^ A frequency of 0.3 Hz and a 10 mm displacement were applied together with a stimulation program divided into an active stimulation period of 16 h, based on 5 min of magnetic stimulation and 25 min of resting time followed by a nonactive period of 8 h, when no magnetic stimulation was applied.^15,33^ Stimulated (S) glass slides and PVDF samples were used as controls for the effect of the magnetic field and the surface charge generated by polarization, respectively. Nonstimulated (NS) surfaces were compared to their stimulated counterparts.

##### 2.4.2.1 Vinculin and Vimentin Immunofluorescence

MSCs were seeded at a density of 2 × 10^3^ cells/cm^2^ in basal medium without FBS. Three replicates per condition were used. After 3 h, the medium was replaced with balanced medium, chosen from previous experiments (described in the Supporting Information), and 1 h later, stimulated samples were placed in the bioreactor. Cells were cultured for 3 days. Stimulated samples were kept in the bioreactor under the stimulation program for the whole duration of the culture. After 3 days, samples were fixed and immunostained following the protocol described in the Supporting Information. Focal adhesions were stained by detecting vinculin using a mouse monoclonal antivinculin antibody (1:400, Sigma-Aldrich, V9264), and vimentin, an intermediate cytoskeleton filament, was detected using a goat polyclonal antivimentin antibody (1:100, Sigma-Aldrich, V4630).

Vinculin was imaged and analyzed as described in the Supporting Information. Also, the number of stress fibers per cell was analyzed using F-actin images. CellProfiler was used to generate a pipeline for their identification and quantification. Focal adhesions and stress fibers were analyzed from at least 25 individual cells per condition from three different replicates.

Vimentin images were acquired using 4 different representative zones per well, using three wells per condition, with an inverted fluorescence microscope EVOS M7000 (Fisher Scientific) and analyzed using CellProfiler. Again, an image processing pipeline was generated to load the DNA (DAPI), F-actin (phalloidin), and vimentin (antibody conjugate Texas Red) for each image set. This was followed by automated detection of cell nuclei, morphology, and marker staining intensity.

##### 2.4.2.2 Metabolomics and Data Analysis

For metabolomics analysis of the electrostimulated MSCs, the cells were cultured as nonstimulated control (nonstimulated on PVDF-CFO), osteogenic control (osteogenic media on glass coverslips), and with stimulation on PVDF-CFO in balanced media. Four replicates per condition were used. After 7 days in the culture, cells were lysed. The cell seeding density and protocol followed are the same as described in Section 2.4.2.1. Stimulated samples were placed in the bioreactor and subjected to the stimulation program for the whole culture duration, 7 days.

After 7 days in culture, the substrates were washed with ice-cold PBS, and cells lysed in metabolomics extraction buffer (water/methanol/chloroform at 1:3:1 ratio) for 60 min with constant agitation at 4 °C. The lysates were then transferred to precooled Eppendorf tubes, centrifuged at 13000*g* at 4 °C for 10 min to remove debris, and the supernatant was stored at −80 °C. The extracts were used for hydrophilic interaction LC/MS analysis (UltiMate 3000 RSLC, Thermo Fisher Scientific), with a 159 × 4.6 mm ZIC- pHILIC column running at 300 μL/min and Orbitrap Exactive (Thermo Fisher Scientific). A standard pipeline, consisting of XCMS (peak picking), MzMatch (filtering and grouping) was used to process the raw mass spectrometry data. Putative metabolites were validated against a panel of standards by mass and predicted retention time. The MetaboAnalyst software (version 4.0) was used to generate heatmaps of selected metabolic maps (see Supporting Information) and PCA plots.

### 2.5 Statistical Analysis

All results are expressed as mean ± standard deviation. Statistical analysis was performed on GraphPad Prism 9 (USA). Samples following a normal distribution, determined by the Shapiro–Wilk test, were analyzed using a two-tailed *t* test or one-way ANOVA for multiple comparisons. The rest of the samples were analyzed by the nonparametric tests, two-tailed Mann–Whitney or Kruskal–Wallis (with Dunn’s multiple comparison test). A 95% confidence interval was set to accept significant intergroup differences (*p* value <0.05).

## 3 Results and Discussion

### 3.1 Film Characterization

**Figure 1:**
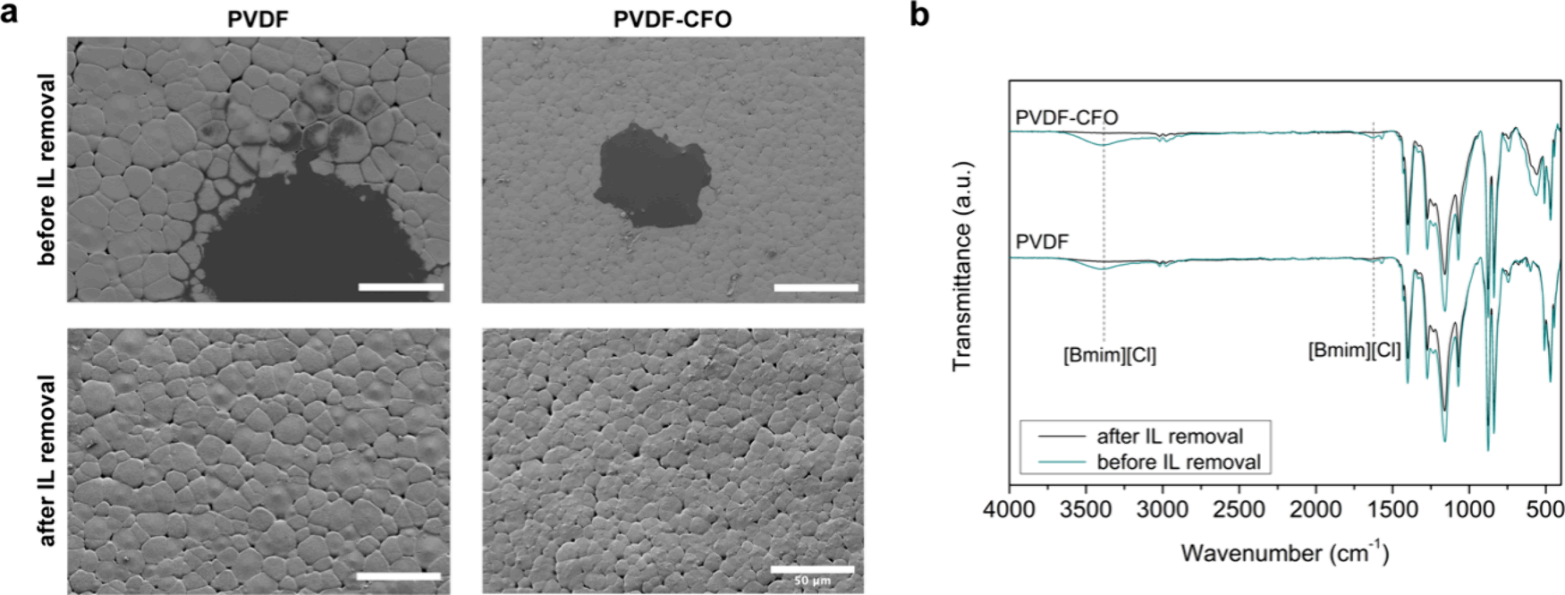
Characterization of PVDF and PVDF-CFO films before and after IL removal. (a) Field emission scanning electron microscopy images of PVDF and PVDF-CFO surfaces before (upper line) and after (lower line) IL was removed by washing. Scale bar 50 μm. (b) FTIR-ATR spectra of PVDF and PVDF-CFO films before (green line) and after (black line) IL removal.

PVDF and PVDF-CFO films were produced by the doctor blade coating technique and crystallized in the presence of the IL [Bmim][Cl]. After crystallization from the melt, IL was removed by several washes with water. As observed in Figure 1a, before [Bmim][Cl] was washed, its presence could be detected on the film surface in the form of dark stains covering PVDF spherulites. This indicates that part of the IL is moved to the sample’s surface by the growth of PVDF crystals. After IL removal (Figure 1a), the dark areas disappear due to the solubility of [Bmim][Cl] in water.^34^ Eliminating the IL allowed for visualizing PVDF’s characteristic spherulitic structure when crystallized from the melt. The spherulites show a slightly smaller size in PVDF-CFO films than in pristine PVDF due to the role of CFO nanoparticles as nucleating agents. The dispersion of these nanoparticles in the initial solution provides a larger number of crystallization nuclei, where polymer spherulites start to grow until they contact a neighbor spherulite.^35^

IL removal was also confirmed by infrared (IR) spectroscopy. PVDF and PVDF-CFO film spectra before and after [Bmim][Cl] elimination are shown in Figure 1b. The characteristic absorption bands of the IL are observed before the removal. A clear peak appears at 3385 cm^–1^, corresponding to the quaternary amine of the [Bmim] cation, and another one at 1635 cm^–1^ from the C=C stretching can also be detected.^36^ After washing, the peaks can no longer be observed, indicating the removal of the IL and supporting the electron microscopy results.

Once the removal of the IL was confirmed, washed films were further characterized. PVDF can present five polymorphs (α, β, γ, δ, and ε), but not all of them are electroactive. α, β, and γ are the most commonly obtained phases by the standard manufacturing techniques, and β is the preferred phase due to having the highest piezoelectric coefficient.^21^ PVDF polymorphs’ vibrational spectra via FTIR-ATR have been validated for phase identification. This approach involves identifying characteristic absorption bands that are unequivocally present in the spectra of one of the phases.^37^ The α-phase is the easiest to identify due to the high number of representative peaks (410, 489, 532, 614, 762, 795, 854, 975, 1149, 1209, 1383, and 1423 cm^–1^), with 762 cm^–1^ typically used to recognize it. Regarding the β-phase, 840 cm^–1^ is usually considered the most characteristic peak. Nevertheless, it has recently been accepted that this band can also contribute from the γ-phase, although this peak tends to appear as a shoulder of the 833 cm^–1^ band for the γ-phase. Any ambiguity can be solved by identifying the absorption peak at 1279 cm^–1^, which unequivocally distinguishes β from γ-phase.^21,37^

**Figure 2:**
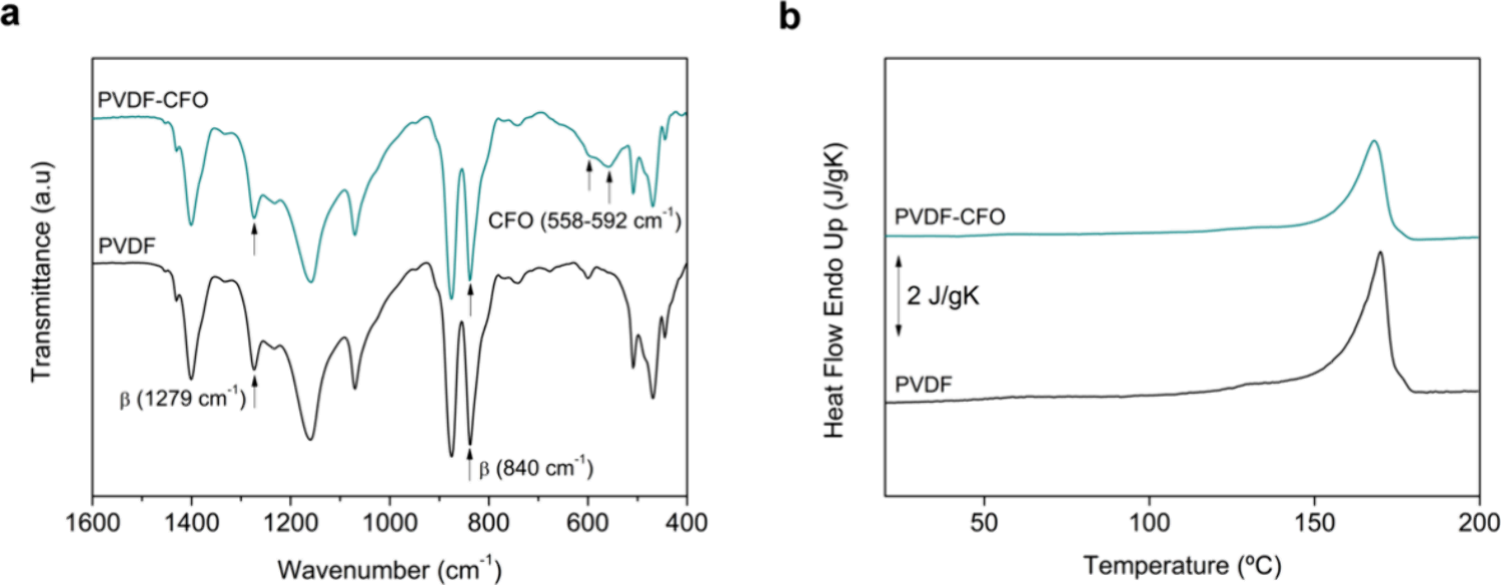
Characterization of PVDF and PVDF-CFO films after IL removal. (a) FTIR-ATR spectra of PVDF and PVDF-CFO films where the β-phase and CFO characteristic peaks are highlighted. (b) DSC heating thermograms of PVDF and PVDF-CFO films.

To ensure the derivation of the most electroactive polymorph, β-phase, PVDF and PVDF-CFO film, FTIR-ATR spectra were analyzed. As shown in Figure 2a, the α-phase identification band at 762 cm^–1^ appears as a shoulder in both spectra, revealing the scarce participation of this phase in the total amount of the crystalline content. PVDF and PVDF-CFO films present a typical β-phase absorption band of 1279 cm^–1^, which corroborates the presence of this polymorph. Moreover, a strong peak at 840 cm^–1^ can be found, which was used to quantify the β-phase percentage in the samples by applying eq 3.^38^
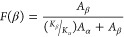
3where *A*_α_ and *A*_β_ are the absorbances at 762 and 840 cm^–1^, corresponding to the α and β phase, respectively, and *K*_α_ (6.1 × 10^4^ cm^2^/mol) and *K*_β_ (7.7 × 10^4^ cm^2^/mol) are the corresponding absorption coefficients of pristine α or β-phase samples.^38^ Quantification revealed that the percentages of β-phase in the PVDF and PVDF-CFO films after IL removal were 95.4 ± 3.4% and 98.6 ± 0.6%, respectively.

Films obtained using a temperature below 70 °C, without a nucleating filler, usually produce β-phase highly porous, fragile, opaque and challenging-to-polarize films. Nevertheless, when the films are produced using higher temperatures (>70 °C) or produced from the melt (*T* ∼ 210 °C) to ensure complete solvent evaporation, as is the case here, they are crystallized mainly in the nonelectroactive α-phase.^21^ Electrically active films can be obtained using the same protocol but incorporating magnetostrictive nanoparticles. CFO nanoparticles are negatively charged, which promotes interaction with the positive CH_2_ charge density of the PVDF chains. This allows the chains’ alignment on the nanoparticle’s surface in the extended all-trans (TTT) conformation, characteristic of the electroactive β-phase.^39^

The β-phase can be induced in neat PVDF films after mechanical stretching of the α-phase material, although the microscopic structure of the films is altered. The characteristic spherulite structure is replaced by a microfibrillar one.^40^ Cell culture assays involving electromechanical stimulation require neat PVDF as a control, where no magnetoelectric effect is observed, regardless of the presence of a magnetic field. Microstructural variations due to stretching provide difficult result interpretation since MSCs can sense diverse nanotopographical cues, which may affect cell behavior.^41^ The introduction of [Bmim][Cl] allows the crystallization of neat PVDF in β-phase, following the same protocol as PVDF-CFO films, without further uniaxial stretching, which alters PVDF microstructure. IL acts similarly to CFO nanoparticles. The interaction between [Bmim][Cl] and the polymer chain between the cation and the CF_2_ groups in the PVDF structure and the anion with CH_2_ leads to the induction of the all-trans planar zigzag β-phase conformation. These results correlate with the ones obtained by Meira et al.,^24^ where PVDF films were manufactured using the same technique and crystallized in the presence of [Bmim][Cl], showing a high content of β-phase, indicating that IL can act as a nucleating agent for this polymorph. Other ILs have been reported as inductors of β-phase crystallization in PVDF substrates, as is the case of 2-hydroxyethyl-trimethylammonium dihydrogen phosphate ([Ch][DHP]) or 1-ethyl-3-methylimidazolium chloride ([Emim][Cl]).^23,24^

It is also worth noting the appearance of characteristic CFO absorption bands at 558 cm^–1^ (Co–O stretching)^42^ and 591 cm^–1^ (Fe–O bond)^43^ in PVDF-CFO films (Figure 2a), confirming the nanoparticles’ incorporation. Their presence was also assessed using a vibrating sample magnetometer that allowed us to calculate the nanoparticle content by applying eq 1.

After IL removal, the thermal properties of the produced films were investigated by using DSC to determine the melting temperature (*T*_m_) and the degree of crystallinity (*X*_c_). Figure 2b shows a single endothermic peak at around 170 °C for both types of films, indicative of polymer melting. *T*_m_ corresponding to PVDF and PVDF-CFO films shows no significant differences, 169.7 and 169.3 °C, respectively. Regarding *X*_*c*_, it was calculated by applying eq 4:
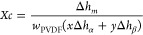
4where Δ*h*_m_ is the melting enthalpy of PVDF and PVDF-CFO films measured by DSC and Δ*h*_α_ and Δ*h*_β_ are the melting enthalpies of a 100% crystalline sample in the α and β phases, whose values are 93.07 J/g and 103.4 J/g, respectively.^44^*w*_PVDF_ is the effective mass fraction of PVDF within the films (provided by their magnetic properties), and *x* and *y* are the percentages of α and β phases present in the sample, obtained by FTIR measurements.

Again, the quantification showed no significant differences in the crystalline content of both samples, 62 ± 0.7% for PVDF and 63.9 ± 3.3% for PVDF-CFO. In this case, the incorporation of CFO does not reduce the degree of crystallinity of the composites by inducing defects during polymer crystallization, as has been reported in related studies.^45,46^

Lastly, the magnetization curve for PVDF-CFO nanocomposites was measured, and it is shown in Figure S2. The room temperature hysteresis loop showed a typical appearance for PVDF-CFO composites^15,47,48^ and a value of magnetization saturation at 18 kOe of 11.1 ± 0.3 emu/g and coercivity of −2776 ± 16 Oe. Quantification showed that the CFO final concentration was 20.4 ± 0.5% (w/w), which, as expected for this manufacturing technique, corresponds to the initial concentration in solution, revealing no CFO loss.

### 3.2 Mesenchymal Stem Cell Response to Electroactive Films at Static Mode

**Figure 3:**
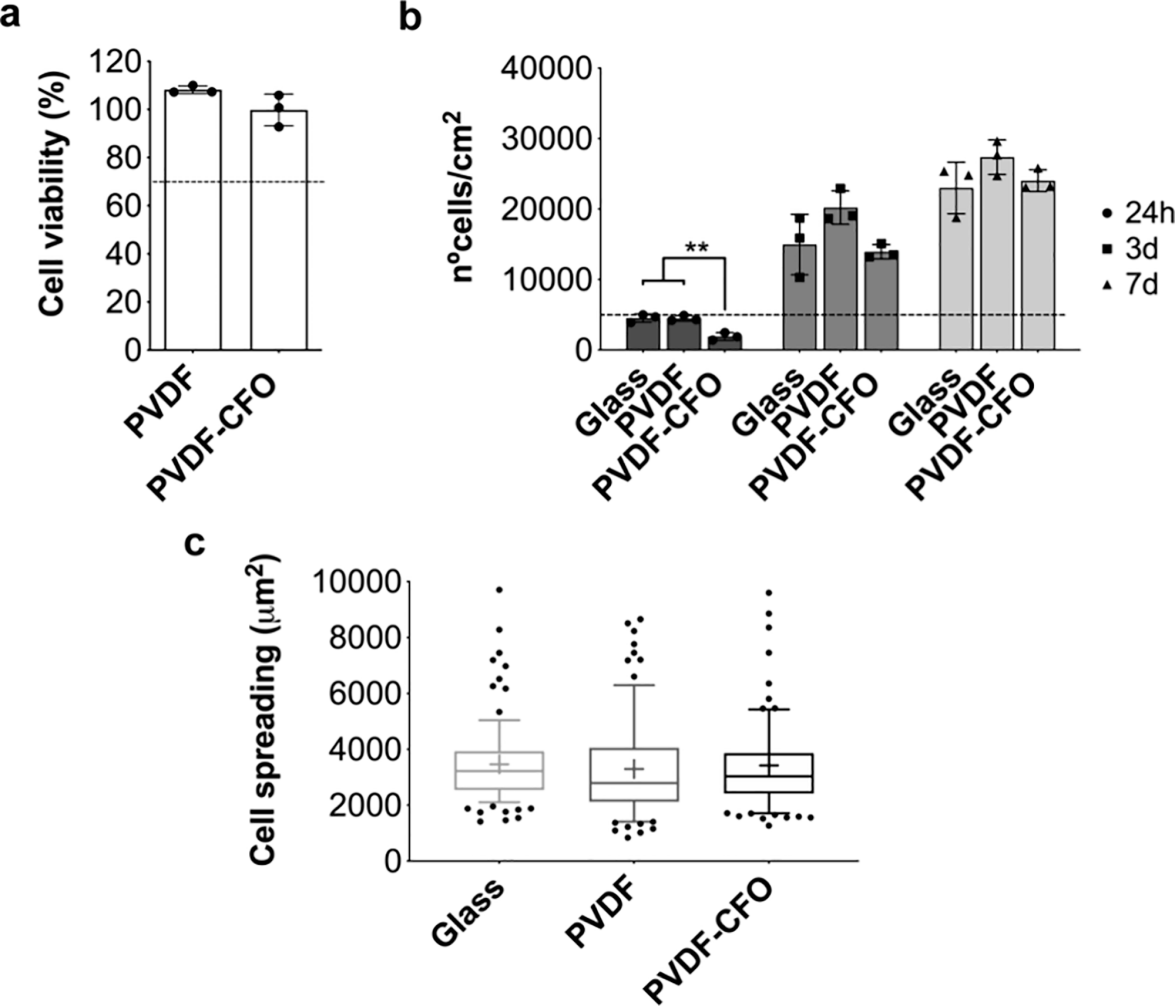
Characterization of initial MSC response on glass, PVDF and PVDF-CFO films after IL removal. (a) Leachable test to determine CFO or possible traces of ionic liquid cytotoxicity. The dashed line corresponds to 70% cell viability, a fixed limit by ISO 10993–5 to consider a biomaterial as cytotoxic. No significant differences are observed (*n* = 3, two-tailed *t* test). b) Cell number per cm^2^ after 1, 3, and 7 days based on MTS test. The dashed line indicates initial seeding density (5 × 10^3^ cells/cm^2^), *p*-value <0.01 (**) (*n* = 3, one-way ANOVA with Holm-Sídák multiple comparison test). (c) Box plot (10–90 percentile) of MSCs cell area measured after 24 h on different cell culture substrates. No significant differences are observed, determined by Kruskal–Wallis with Dunn’s multiple comparison test. 85 cells per condition, at least, from three different replicates were used for the analysis.

MSCs’ initial behavior at static mode without electromechanical stimulation was tested. The first step was to prove the absence of cytotoxicity of possible IL traces or CFO nanoparticles nonincorporated into the polymer matrix. Biocompatibility of the films was studied using an indirect cytotoxicity assay or leachable test, where PVDF and PVDF-CFO films were placed in contact with a basal medium for 24 h. MSC viability after 48 h in contact with the conditioned medium was analyzed (Figure 3a), revealing no toxicity due to leaching. FTIR-ATR also suggests a Lack of IL leaching (Figure 1b).

The correct incorporation of the nanoparticles into the polymer matrix does not rule out that the presence of CFO exposed on the film surface may affect MSCs’ initial adhesion and proliferation. FTIR-ATR spectra revealed the existence of magnetostrictive nanoparticles close to or on the film surface, as demonstrated by their characteristic absorption bands in the composite spectra (Figure 2a). As shown in Figure 3b, after 24 h, there is a significant difference between the number of cells on glass and PVDF substrates compared to PVDF-CFO, where the cell count is lower than the initial seeding density (dashed line). The presence of exposed CFO nanoparticles may hinder initial MSC adhesion. Nevertheless, after 3 days, cell count on PVDF-CFO substrates shows no significant differences compared to the other conditions, a trend maintained after 7 days; indeed, MSCs show a proliferative pattern on PVDF-CFO catching up with control substrates by day 3. Even though cell count was significantly lower after 24 h in PVDF-CFO substrates, cell spreading analysis revealed that attached cells display similar cell area compared to glass slide control (Figure 3c). Cell spreading shows no significant differences between conditions, which indicates that even if the cell count is lower in PVDF-CFO substrates after 24 h, MSCs are well attached.

### 3.3 Mesenchymal Stem Cell Response to Piezoelectric Stimulation at the Cytoskeleton Level

After selecting the appropriate medium in static cell culture experiments (see Supporting Information), MSCs were seeded on different cell culture supports and stimulated using a magnetic bioreactor,^32^ applying a stimulation program based on reproducing daily human activity (active stimulation of 16 h and nonactive period of 8 h, see details in materials and methods section). Cells were cultured for 3 days, and focal adhesions, actin stress fibers, and intermediate filaments were studied to determine how electromechanical stimulation affects cytoskeleton dynamics.

**Figure 4:**
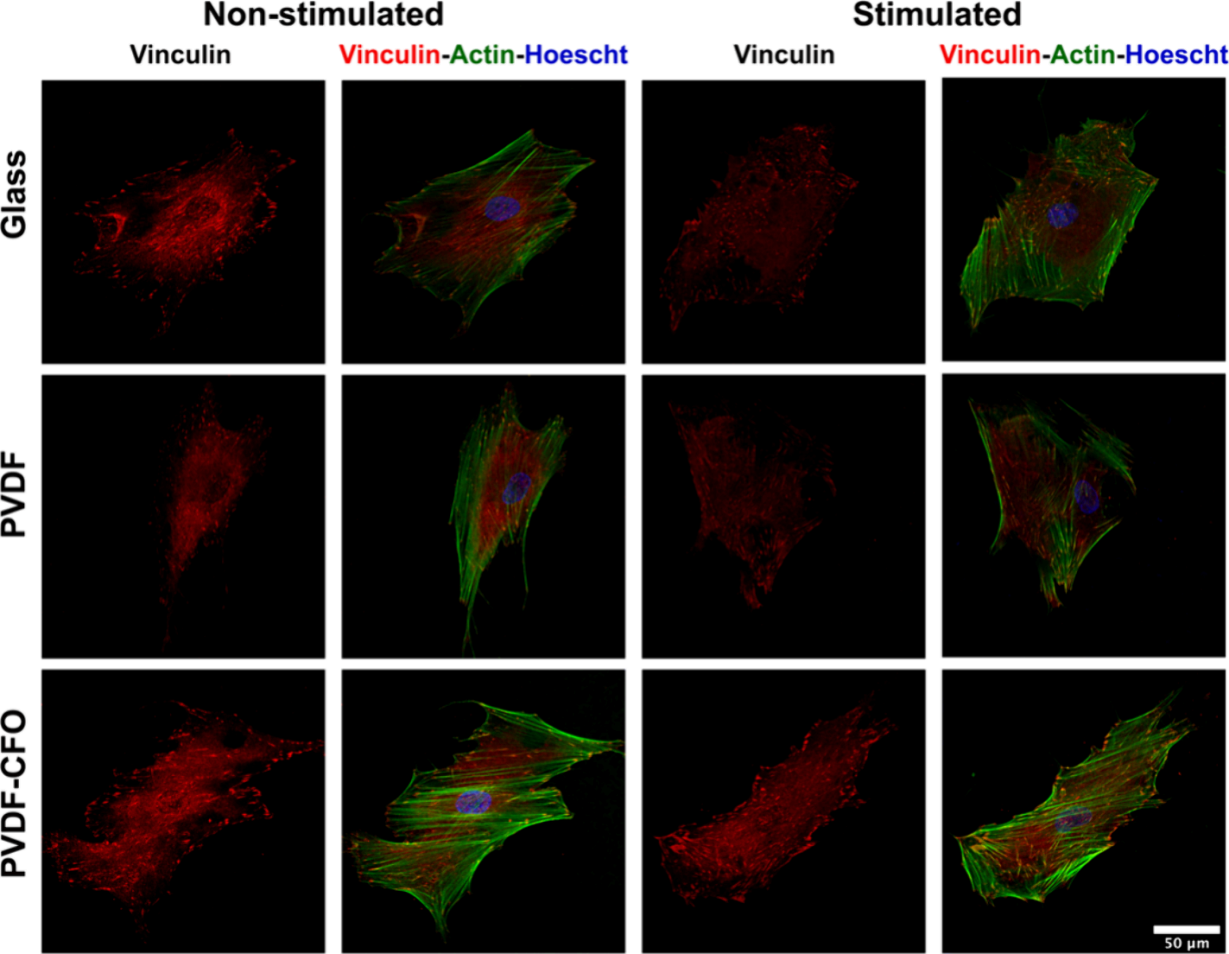
Representative fluorescence images of MSCs after 3 days cultured on glass, PVDF, and PVDF-CFO in static mode (nonstimulated (NS)) or dynamic (stimulated (S)) conditions (vinculin (red), F-actin (green), and nuclei (Hoechst-blue); scale bar: 50 μm).

**Figure 5:**
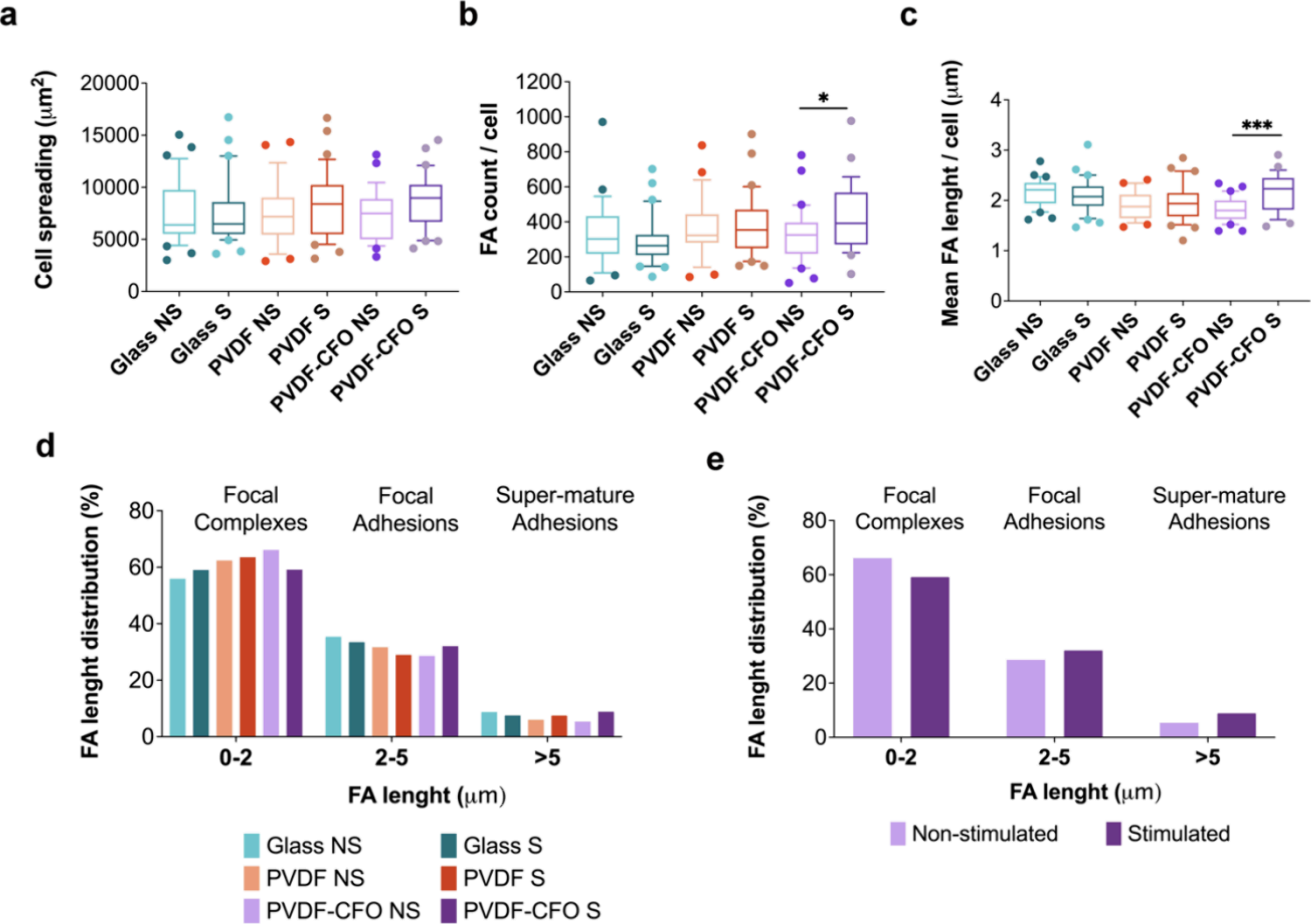
Focal adhesion (FA) and cell area analysis of MSCs after 3 days cultured on glass, PVDF and PVDF-CFO in static (nonstimulated (NS)) or dynamic (stimulated (S)) conditions. Box and whiskers (10–90 percentile) of (a) cell area, (b) the number of FA per cell, and (c) the mean FA length per cell. (d) Histogram of FA length distribution (%) of stimulated and nonstimulated MSCs cultured on glass, PVDF, and PVDF-CFO classified according to FA length in focal complexes (0–2 μm), focal adhesions (2–5 μm), or super mature focal adhesions (>5 μm). (e) Histogram of FA length distribution (%) of stimulated and nonstimulated MSCs cultured on PVDF-CFO films following the same classification. Statistical differences between cells cultured on the same support (glass, PVDF, or PVDF-CFO) in static and dynamic conditions were determined by a two-tailed *t* test when following a normal distribution (Shapiro–Wilk test). If not, a two-tailed Mann–Whitney test was used. *p* value legend: *p* < 0.05 (*), *p* < 0.01 (**), *p* < 0.001 (***). Twenty-five individual cells per condition, at least, from three different replicates were used for all the analysis.

Figure 4 shows representative fluorescence images of the F-actin cytoskeleton and vinculin immunostaining of nonstimulated and stimulated MSCs. Cells show a well-developed cytoskeleton and a spindle-shaped morphology under every condition, which is characteristic of these multipotent cells. Those images were used to study the MSC response to piezoelectric stimulation. Cell spreading was again quantified, and the results presented in Figure 5a show no significant differences between the studied conditions, regardless of the cell culture substrate or the presence of a magnetic field applied by the bioreactor. In the case of PVDF-CFO supports, the magnetic field induces electromechanical stimulation,^33^ which is transmitted to the MSCs cultured on the surface. Nevertheless, this cue did not affect the cell spreading area.

Focal adhesions were also quantified, and FA count and mean FA length per cell were compared between nonstimulated and stimulated cell culture supports. As can be seen in Figure 5b,c, there are significant differences between PVDF-CFO NS and S. These differences cannot be seen in PVDF and glass slides, where the application of a magnetic field generates no piezoelectric stimulation due to the absence of the CFO nanoparticles. The electromechanical cue provided by combining a piezoelectric matrix and a magnetostrictive component enhances the appearance of FA and their mean length. These results can be seen when FA length is classified into focal complexes (0–2 μm), focal adhesions (2–5 μm), and supermature focal adhesions (>5 μm) (Figure 5d,e). Nonstimulated PVDF-CFO samples present an increase in the frequency of focal complexes. On the other hand, stimulated PVDF-CFO samples show a higher number of focal adhesions ranging from 2 to 5 μm and supermature adhesions compared to PVDF-CFO NS. As mentioned, increased focal adhesion length indicates MSC osteogenesis.^19^ Longer FA support more contractile morphologies and show higher intracellular tension.^49,50^ MSCs display small and transient adhesions in the stem cell niche, which allows them more dynamic interactions with the extracellular matrix, which is fundamental for MSC self-renewal.^51^ This concept was demonstrated using nanotopography, where MSCs cultured on nanotopographies promoting the formation of smaller FA retained multipotency, whereas nanotopographies promoting larger FA formed osteogenesis.^52,53^

Changes in cytoskeleton tension and reorganization, correlated with the increase in length of focal adhesions, can be observed when MSCs are subjected to physical cues that induce osteogenesis.^54^ The number of stress fibers per cell was evaluated to elucidate cytoskeletal response to MSCs with electromechanical stimulation.

**Figure 6:**
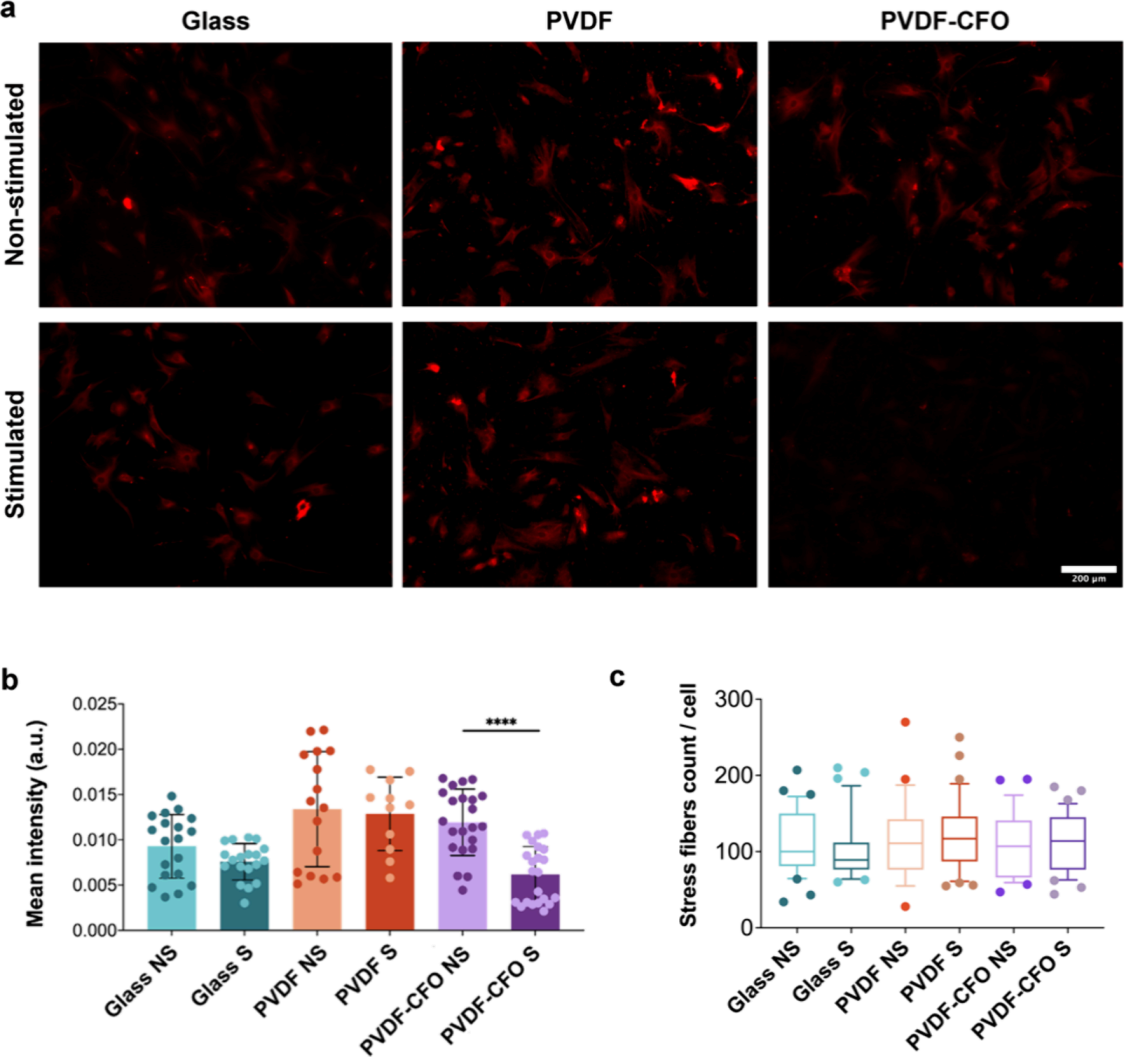
Vimentin analysis of MSCs after 3 days cultured on glass, PVDF, and PVDF-CFO in static (nonstimulated (NS)) or dynamic (stimulated (S)) conditions. (a) Representative fluorescence images of vimentin immunofluorescence. Scale bar: 200 μm. (b) Quantification of vimentin mean intensity on different cell culture supports. Four representative zones per well were analyzed from at least 3 replicates. Statistical differences were determined by Kruskal–Wallis with Dunn’s multiple comparison test. *p* value <0.0001 (****). (c) Box and whiskers (10–90 percentile) of the number of actin stress fibers per cell in MSCs cultured on glass, PVDF and PVDF-CFO in static (nonstimulated (NS)) or dynamic (stimulated (S)) conditions after 3 days.

A clear effect could be seen on the intermediate filament vimentin upon piezoelectric stimulation. Figure 6a displays characteristic images of vimentin immunostaining in MSCs after 3 days in culture under the influence or absence of a magnetic field applied by the bioreactor. Quantification (Figure 6b) reveals a significant decrease in vimentin intensity in PVDF-CFO stimulated samples compared to nonstimulated PVDF-CFO. On the contrary, Figure 6c shows that this stimulation does not significantly change the actin stress fiber count per cell.

A reduction in vimentin expression could be correlated with MSCs’ osteogenic differentiation. The reduction in vimentin expression during differentiation has been described in both MC3T3-E1 osteoblasts and bone marrow stromal progenitors, with vimentin mRNA levels decreasing as differentiation progresses.^55,56^

Fan et al.^56^ explored the changes in the spatial distribution of vimentin and actin stress fibers during MSCs’ osteogenic differentiation. During MSC osteogenesis, a reduction in vimentin intensity was observed and correlated with vimentin losing part of its cytoplasmatic space and being replaced by actin stress fibers. Vimentin was restrained to the top of the cells, away from the nucleus and ventral side, and its network became smaller. On the other hand, Lian et al.^55^ confirmed that vimentin down-regulation during osteoblast differentiation is required to relieve its inhibition of activating transcription factor 4 (ATF4). ATF4 determines osteoblasts’ initiation and terminal differentiation by triggering osteocalcin expression synergistically with Runx2/Cbfa.^57^ Vimentin binds to ATF4 in osteoblasts, preventing its differentiation and ATF4-dependent osteocalcin transcription. During osteogenesis, MSCs experience cytoskeletal remodeling; vimentin is replaced by actin filaments, leading to ATF4 transcription and subsequent osteocalcin expression. The reduction in vimentin expression in PVDF-CFO stimulated samples may lead to the activation of ATF4, indicating an early sign of the MSC commitment to the osteogenic lineage. In fact, it has been previously reported that MSCs respond to osteogenic inductive physical cues by reorganizing vimentin. MSCs cultured on nanopatterned surfaces promoting osteogenic differentiation showed a significantly reduced vimentin density, which was correlated with alterations in the packing of chromosome territories and changes in transcription factor activity.^52^

### 3.4 Mesenchymal Stem Cell Response to Piezoelectric Stimulation at the Metabolite Level

**Figure 7:**
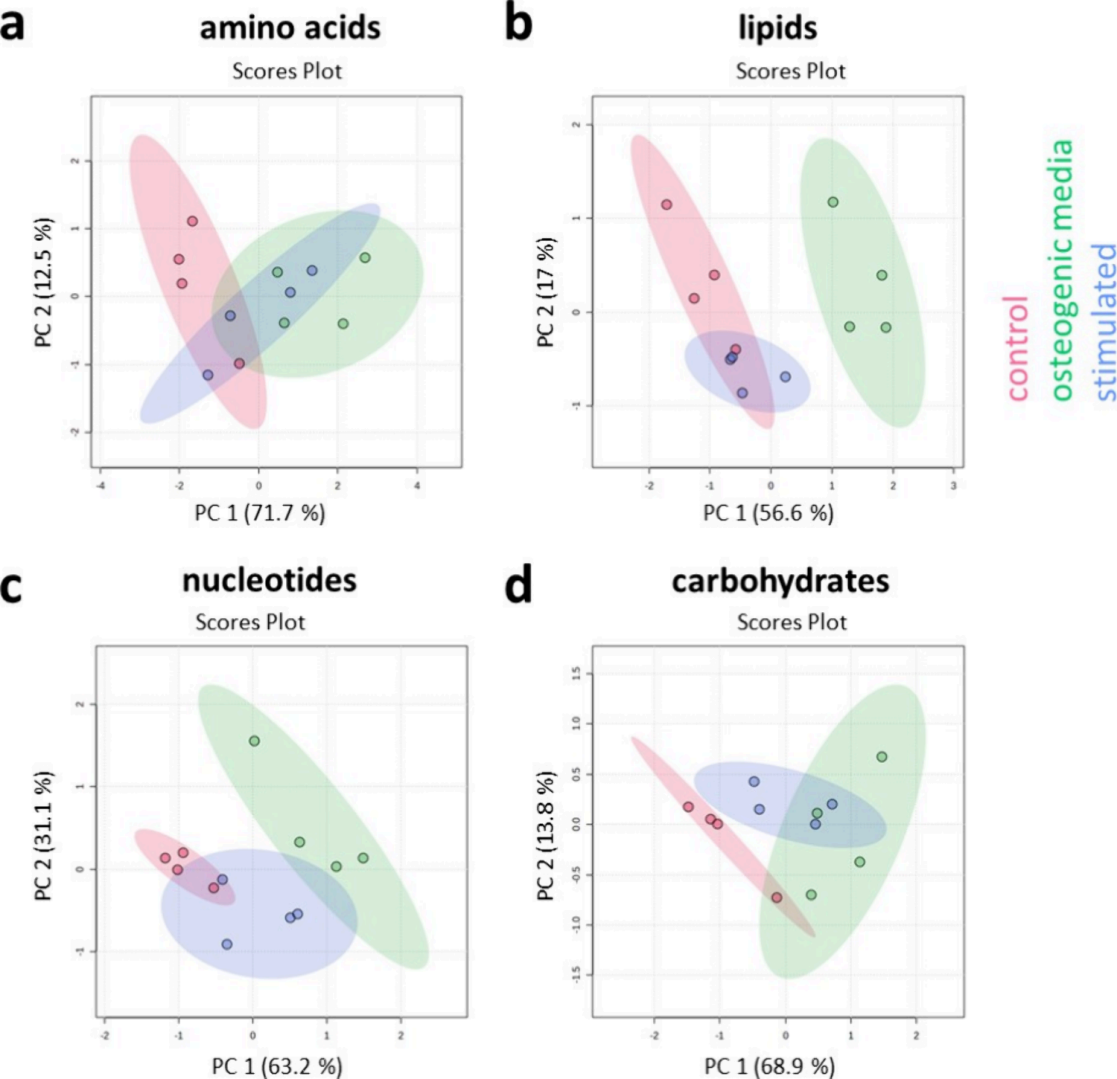
Metabolomic analysis of MSCs cultured on nonstimulated control (nonstimulated on PVDF-CFO), osteogenic control (osteogenic media on glass slides), and with stimulation on PVDF-CFO in balanced media for 7 days. Data presented as principal component analysis, the separation of the two control conditions (nonstimulated and osteogenic) could be clearly observed (*x* axis: PC1, *y* axis: PC2). Trends for the stimulated MSC populations showed an osteogenic profile for (a) amino acids and an intermediate metabolic profile for (b) lipids, (c) nucleotides, and (d) carbohydrates (in this last case two samples overlap with the osteogenic control). Four replicates were used to generate the data.

Next, we cultured MSCs for 7 days in nonstimulated control (nonstimulated on PVDF-CFO), osteogenic control (osteogenic media on glass slides), and with stimulation on PVDF-CFO in balanced media. At the end of the culture, metabolites were isolated, and mass spectrometry analysis of metabolites was performed. We looked at amino acid, lipid, nucleotide, and carbohydrate metabolism data sets as MSC differentiation has been linked to changes in cell energetics.^58^ Using principal component analysis, it was clear that nonstimulated control and osteogenic control separated significantly along principal component 1 (*x*-axis) for all data sets (Figure 7). For stimulated MSCs, the metabolite expression pattern of the cells grouped in between nonstimulated control and osteogenic control (Figure 7). Amino acid expression of the stimulated group almost overlaps with the osteogenic control (Figure 7a), and carbohydrate expression of two of the four replicates coincides with the osteogenic control (Figure 7d). The results seem to indicate that electromechanical stimulation tends to induce the osteogenic phenotype of MSCs at the level of the metabolites.

Altogether, the evidence presented here indicates that MSCs can respond to electromechanical stimulation on 2D substrates using focal adhesions. The lengthening of adhesions leads to a cytoskeleton response, where the vimentin density is decreased. It also leads to the rearrangement of energy metabolism toward an osteogenic phenotype.

As previously reviewed, piezoelectric stimulation activates different signaling pathways that converge in the expression of osteogenic-related genes.^59^ These cascades are not independent; they tend to overlap at different cell levels, revealing that their boundaries are not tight.

Besides the effect of piezoelectric stimulation on vimentin rearrangement leading to ATF4 transcription, piezoelectric substrates have an associated surface potential that can alter the conformation of proteins adsorbed from either the cell culture media or surface coatings. This change in conformation affects the exposure of the protein’s cell adhesion domains.^31^ Integrins bind extracellular matrix proteins, activating mechanotransduction signaling pathways. They mediate the response to piezoelectric stimulation by activating focal adhesion kinase (FAK). When FAK is recruited to focal adhesions through cytoskeletal anchor proteins, such as talin and paxillin, the clustered FAK molecules undergo phosphorylation, creating a phosphotyrosine docking site for Src family proteins. These Src proteins bind growth factor receptor-bound protein 2/son of sevenless (Grb2/SOS), triggering ERK activation. ERK activation ultimately leads to the transcription of RUNX2, driving MSC osteogenic differentiation.^60^

Besides integrin-mediated responses, intracellular calcium oscillations have also been described as potential drivers of MSC differentiation for cells cultured on piezoelectric substrates. Voltage-gated calcium channels (VGCC) facilitate the influx of Ca^2+^ into the cell, increasing its concentration in the cytoplasm. Liu et al. demonstrated a link between this rise in cytoplasmic calcium and p38 phosphorylation after piezoelectric stimulation.^61^ Phosphorylation of p38 promotes the expression of osterix, which in turn regulates the expression of various osteogenic factors, including osteonectin, osteopontin, osteocalcin, and alkaline phosphatase.^62^

Previous reports have explored the effect on cell adhesion and proliferation under higher magnetic fields than in our study.^63^ However, we reproduced the conditions in ref (64) for being adequate for the osteogenic differentiation of preosteoblasts.

Our study demonstrates that the piezoelectric stimulation induces the osteogenic differentiation of mesenchymal stem cells, and future studies should focus on demonstrating that these primed cells are better candidates for the regeneration of bone than nonpre-differentiated mesenchymal stem cells.

## 4 Conclusions

PVDF and PVDF-CFO electroactive films were produced by the doctor blade coating technique in the β-phase. The crystallization of PVDF in the presence of the IL [Bmim][Cl] allowed the induction of its most electroactive phase, even in neat PVDF films, without further uniaxial stretching. After crystallization, several washes with water successfully removed the IL. Films proved biocompatible, allowing MSC adhesion and proliferation in the short term compared to glass slide controls. The incorporation of magnetostrictive nanoparticles allowed the electromechanical stimulation of MSCs to elucidate their response regarding cytoskeleton dynamics. The balanced medium was combined with electromechanical stimulation, showing that stimulated PVDF-CFO surfaces improved focal adhesion length compared to PVDF-CFO nonstimulated supports. Also, the vimentin density was decreased in the presence of piezoelectric stimulation. A rearrangement of energy metabolism toward an osteogenic phenotype was observed under piezoelectric stimulation. These results prove that MSCs respond to this kind of stimulation using focal adhesions, cytoskeleton reorganization, and metabolite expression. This opens the way for further studies of MSC fate determination using piezoelectric cell culture supports. As future perspectives, we could deeply analyze the differentiation pathways that are activated in mesenchymal stem cells submitted to the electromechanical stimulation.

## Supporting Information Available

Vbrating sample magnetometer curve of the PVDF/CFO sample; the materials, methods, and results and discussion for the medium selection at static cell culture mode; heatmaps of the metabolomics analysis performed on cells after the dynamic cell culture experiments (PDF)

## ABREVIATIONS

ATF4: activating transcription factor 4
ATR: attenuated total reflection
[Bmim][Cl]: 1-butyl-3-methyl-imidazolium chloride
BSA: bovine serum albumin
CFO: cobalt ferrite oxide
DMEM: Dulbecco’s modified Eagle medium
DPBS: Dulbecco’s phosphate buffered saline
DSC: differential scanning calorimetry
ERK1/2: extracellular signal-related kinase
FA: focal adhesions
FESEM: field emission scanning electron microscopy
FTIR: Fourier transform infrared spectroscopy
ILs: ionic liquids
MAPK: mitogen-activated protein kinases
MSCs: mesenchymal stem cells
MTS: 3-(4,5-dimethylthiazol-2-yl)-5-(3-carboxymethoxyphenyl)-2-(4-sulfophenyl)-2H-tetrazolium
NEAA: nonessential amino acids
NS: nonstimulated
P/S: penicillin/streptomycin
PVDF: poly(vinylidene) fluoride
RUNX2: runt-related transcription factor 2
S: stimulated
*T*_m_: melting temperature
VSM: vibrating sample magnetometer
*X*_c_: degree of crystallinity
