# Supplementary material for: Piezoelectric Stimulation Induces Osteogenesis in Mesenchymal Stem Cells Cultured on Electroactive Two-Dimensional Substrates
Source: ACS Appl Polym Mater. 2024 Nov 6;6(22):13710–22. doi: 10.1021/acsapm.4c02485 (PMC11590054; doi:10.1021/acsapm.4c02485)
Supplement: Supplementary file 1 — ap4c02485_si_001.pdf [file ap4c02485_si_001.pdf]

Supporting information

Piezoelectric stimulation induces osteogenesis in mesenchymal stem cells cultured on electroactive 2D substrates

Maria Guillot-Ferriols[1,2], Carlos M. Costa[3,4], Daniela M. Correia[5], José Carlos Rodríguez-Hernández[1], Penelope M. Tsimbouri[6], Senentxu Lanceros-Méndez[3,7,8], Matthew J. Dalby[6], José Luis Gómez Ribelles[1,2], Gloria Gallego-Ferrer[1,2*]*

[1] Center for Biomaterials and Tissue Engineering (CBIT), Universitat Politècnica de València, 46022 Valencia, Spain

[2] Biomedical Research Networking Center on Bioengineering, Biomaterials and Nanomedicine (CIBER-BBN), 46022 Valencia, Spain

[3] Physics Centre of Minho and Porto Universities (CF-UM-UP) and Laboratory of Physics for Materials and Emergent Technologies, LapMET, University of Minho, 4710-057 Braga, Portugal

[4] Institute of Science and Innovation for Bio-Sustainability (IB-S), University of Minho, 4710-057 Braga, Portugal

[5] Center of Chemistry, Universidade Do Minho, 4710-058 Braga, Portugal

[6] Center for the Cellular Microenvironment, School of Molecular Biosciences, College of Medical, Veterinary and Life Sciences, University of Glasgow, Glasgow, G12 8QQ, United Kingdom.

[7] BCMaterials, Basque Center for Materials, Applications and Nanostructures, UPV/EHU Science Park, 48940 Leioa, Spain

[8] IKERBASQUE, Basque Foundation for Science, 48009 Bilbao, Spain

CORRESPONDING AUTHORS

* José Luis Gómez Ribelles. Center for Biomaterials and Tissue Engineering (CBIT), Universitat Politècnica de València, Camino de vera s/n, 46022-Valencia (Spain). Phone: +34 96 3877275; Fax: + 34 96 3877329. ORCID: 0000-0001-9099-0885. E-mail: jlgomez@ter.upv.es

* Gloria Gallego-Ferrer. Center for Biomaterials and Tissue Engineering (CBIT), Universitat Politècnica de València, Camino de vera s/n, 46022-Valencia (Spain). Phone: +34 96 3877324; Fax: + 34 96 3877329. ORCID: 0000-0002-2428-0903. E-mail: ggallego@ter.upv.es

MATERIALS AND METHODS

Medium selection at static mode for subsequent piezoelectric stimulation

To choose the appropriate medium for subsequent differentiation experiments, focal adhesions were analysed using diverse media formulations in static conditions (no stimulation applied). MSCs were seeded on PVDF, PVDF-CFO, and glass slides at a density of 2 x 10^3 cells/cm^2 in basal medium without FBS. After 3 h, media was replaced for complete basal medium (containing FBS), osteogenic medium (dexamethasone 100 nM, Ascorbate-2-phosphate 200 µM and b-glycerophosphate disodium salt hydrate 10 mM) or balanced medium, a mixture 1:1 (v/v) of osteogenic and adipogenic media (dexamethasone 1 µM, 3-isobutyl-1-methylxanthine 500 µM, insulin 1,72 µM and indomethacin 100 µM)[1].

After 3 days, cells were fixed, permeabilised and blocked following the protocol described in section 2.4.1.2 of the main text of the manuscript. Then, the samples were incubated with mouse monoclonal anti-vinculin antibody (1:400, Sigma Aldrich, V9264) in conjunction with ActinGreen 488 ReadyProbes reagent (AlexaFluor 488 Phalloidin, Fisher Scientific) in blocking buffer overnight at 4 °C. After washing three times for 5 min with DPBS/Tween-20

0.1 % (v/v), cells were incubated with a biotinylated anti-mouse secondary antibody (1:50, Vector Laboratories, BA-2000) for 1 h at 37 °C. After washing, Texas Red conjugated streptavidin (1:50, Vector Laboratories, SA-5006) was added to the samples and incubated for 30 min at 4 °C followed by washing and mounting using fluoroshield mounting medium with DAPI (Abcam).

Samples were imaged using a confocal microscope (Zeiss LSM 880 Confocal Microsystem). Individual cells were analysed using CellProfiler. Briefly, an image processing pipeline was generated to load the DNA (DAPI), F-actin (phalloidin) and vinculin (antibody conjugate Texas Red) for each image set. This was followed by automated detection of cell nuclei, cell morphology and detection of focal adhesions. Focal adhesion number per cell, mean focal adhesion length per cell and focal adhesion length distribution were quantified. The pipeline and an example of the identification of focal adhesions performed using CellProfiler are shown in Figure S1.

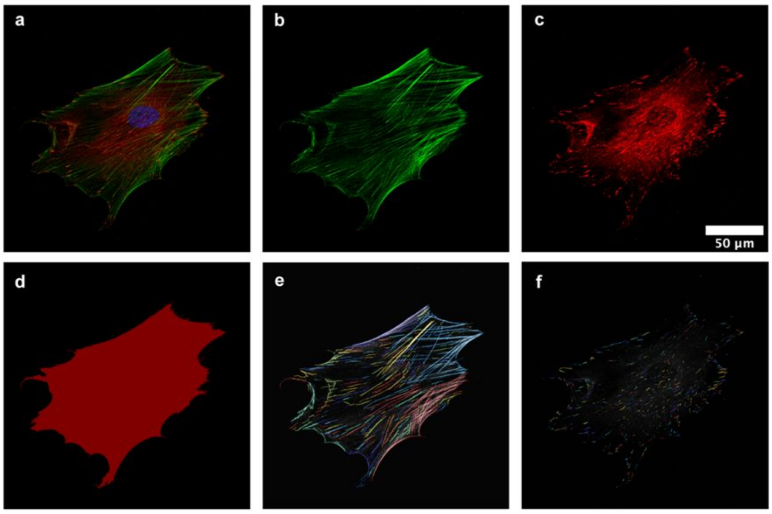

Figure S1. Identification and quantification of focal adhesions, stress fibres and cell spreading using CellProfiler software. a) Merged immunofluorescence image of vinculin (red), F-actin (green) and nucleus (blue). b) F-actin cytoskeleton. c) Vinculin immunofluorescence. d) The masked cell area was obtained after processing F-actin images. e) Image overlay of identified

stress fibres and F-actin. f) Image overlay of identified focal adhesions and vinculin immunofluorescence. Scale bar 50 µm.

RESULTS AND DISCUSSION

VSM curve of the PVDF/CFO sample

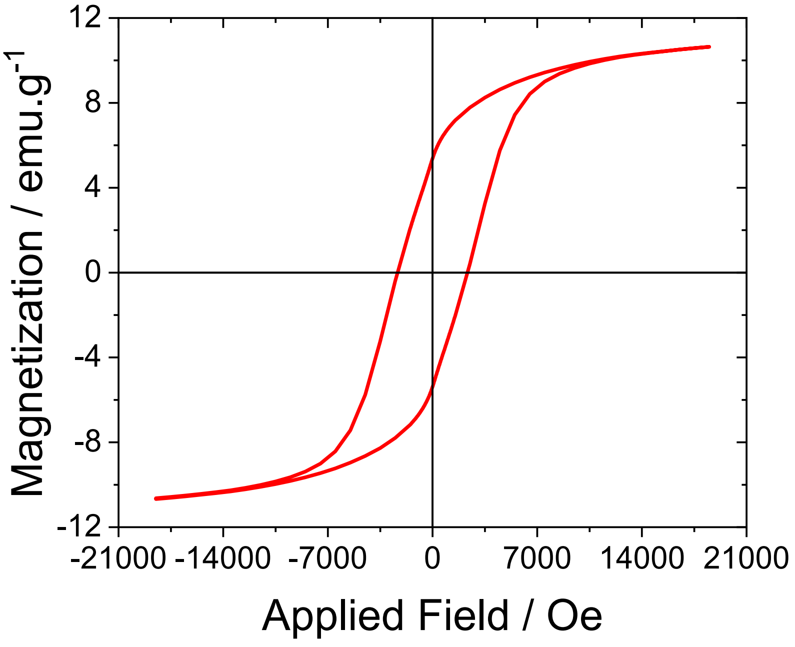

Figure S2. Room-temperature magnetisation curve of PVDF-CFO films

Medium selection for piezoelectric stimulation

Cell culture medium plays a fundamental role in the differentiation process when combined with electromechanical stimulation. The appropriate cell culture medium enhances the stimulation effect on MSCs' osteogenic differentiation. Therefore, different media were tested in static conditions to study their impact on cell area and the formation of focal adhesions, which are crucial during MSCs' osteogenic differentiation process.

Cell spreading area in different substrates and media formulations (basal, osteogenic and balanced) was analysed after 3 days in culture, as seen in Figure S3a. The balanced medium

was also included because the 1:1 mixture of osteogenic and adipogenic media helps MSCs to differentiate in response to other cues, such as piezoelectric stimulation[1]. Contrary to expected, the balanced medium showed a higher cell spreading area in all the substrates, including the glass slide control. However, differences were only significant in PVDF films (Figure S3a). We note that larger cell spreading areas benefit the osteogenic differentiation of MSCs and the maintenance of their differentiated phenotype[2,3].

The focal adhesion analysis also followed this trend. Using different cell media formulations, cells showed increased FA number and FA mean length per cell cultured with balanced media. Again, differences in FA count per cell were only significant in PVDF surfaces (Figure S3b). Nevertheless, the trend was observed on the other surfaces. When analysing FA mean length per cell, differences were significant for glass, PVDF and PVDF-CFO when comparing cells cultured with balanced and basal media. Balanced medium favours the formation of longer FA, as can be seen in Figure S3c. Again, larger adhesions are thought to be important for enhanced osteogenesis[4,5].

Finally, measured FA were represented as focal adhesion distribution according to their length on different substrates and cell media following the classification established by Biggs *et al.*[6] Structures measuring less than 2 μm were assigned as focal complexes; those from 2 to 5 μm were designated as focal adhesions, while those over 5 μm long were classified as super mature adhesions. Focal complexes appear as dot-like structures around 1 μm long, which evolve into focal adhesions due to the intracellular and extracellular tension when integrin packing density can increase by a factor of three-fold. Matured FAs are typically dashed-shaped, 2-5 μm, and contain vinculin, paxillin and talin. As Biggs *et al.* demonstrated, osteoblasts require longer focal adhesion formation, which increases intracellular tension linked to osteogenesis[6].

As shown in Figure S3d, MSCs cultured on glass, PVDF and PVDF-CFO using basal medium demonstrated an increase in the frequency of focal complexes in the range of 0 to 2 μm

compared to balanced and osteogenic media. Nevertheless, when cells were cultured in balanced and osteogenic media, they possessed significantly increased numbers of focal adhesions measuring between 2 and 5 µm. Although the number of super mature adhesions (>5 µm) was not superior to 10 % of the total amount, the trend observed for focal adhesions was maintained, with an increase in balanced and osteogenic media.

Taking all these results together, balanced medium was selected for subsequent differentiation experiments under dynamic conditions (see main text in the manuscript). We propose that balanced medium provides favourable cues for MSCs osteogenic differentiation, as demonstrated by the increased cell area, number, and length of FA. Moreover, exposure to adipogenesis and osteogenesis-promoting soluble cues does not condition MSCs to choose a differentiation pathway; instead, it provides the right stimuli to guide them in combination with electromechanical stimulation.

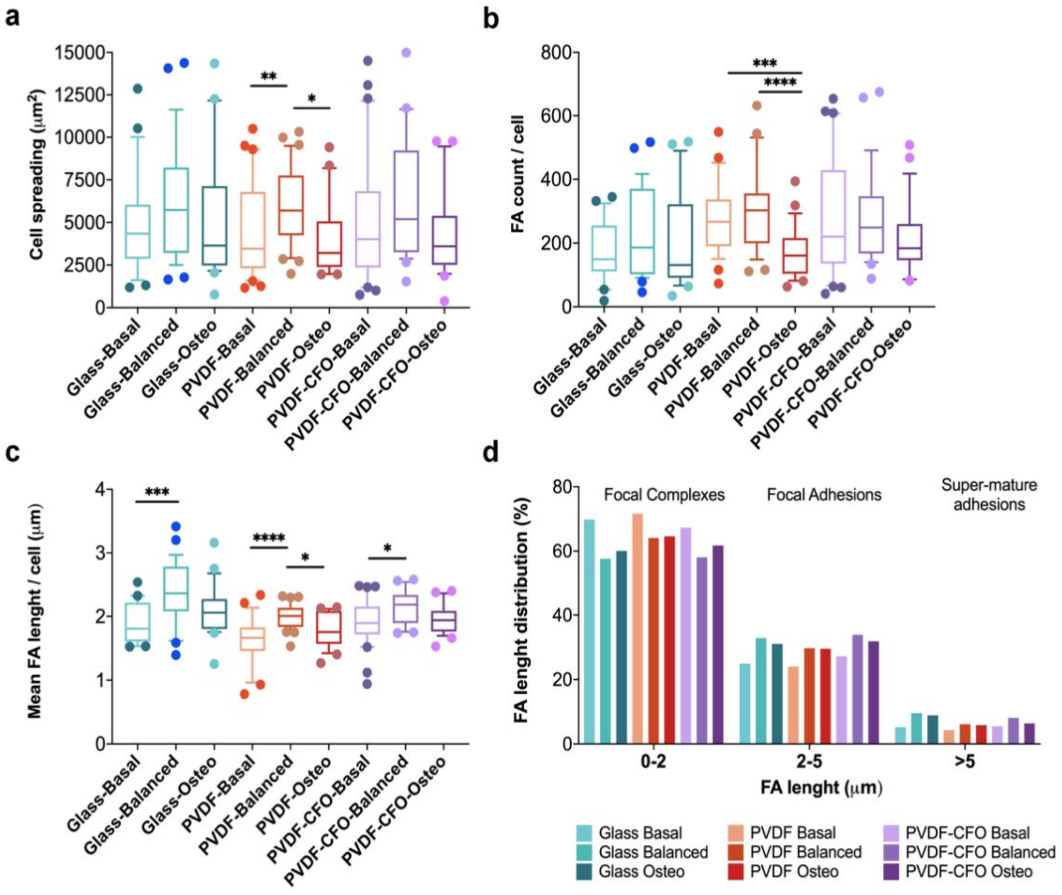

Figure S3. Focal adhesion (FA) and cell area analysis of MSCs after 3 days, cultured in different media formulations and cell culture supports. Box plot (10-90 percentile) of MSCs a) cell area, b) number of FA per cell and c) mean FA length per cell. d) Histogram of FA length distribution (%) classified according to FA length in focal complexes (0-2 μm), focal adhesions (2-5 μm) or super mature focal adhesions (>5 μm). Statistical differences between cells cultured in the same support with different media formulations were determined by non-parametric Kruskal-Wallis and Dunn's multiple comparison test. p-value legend: $p < 0.05$ (*), $p < 0.01$ (**), $p < 0.001$ (***), $p < 0.0001$ (****). 20 individual cells per condition, at least, from three different replicates were used for all the analysis.

Metabolomic analysis heatmaps

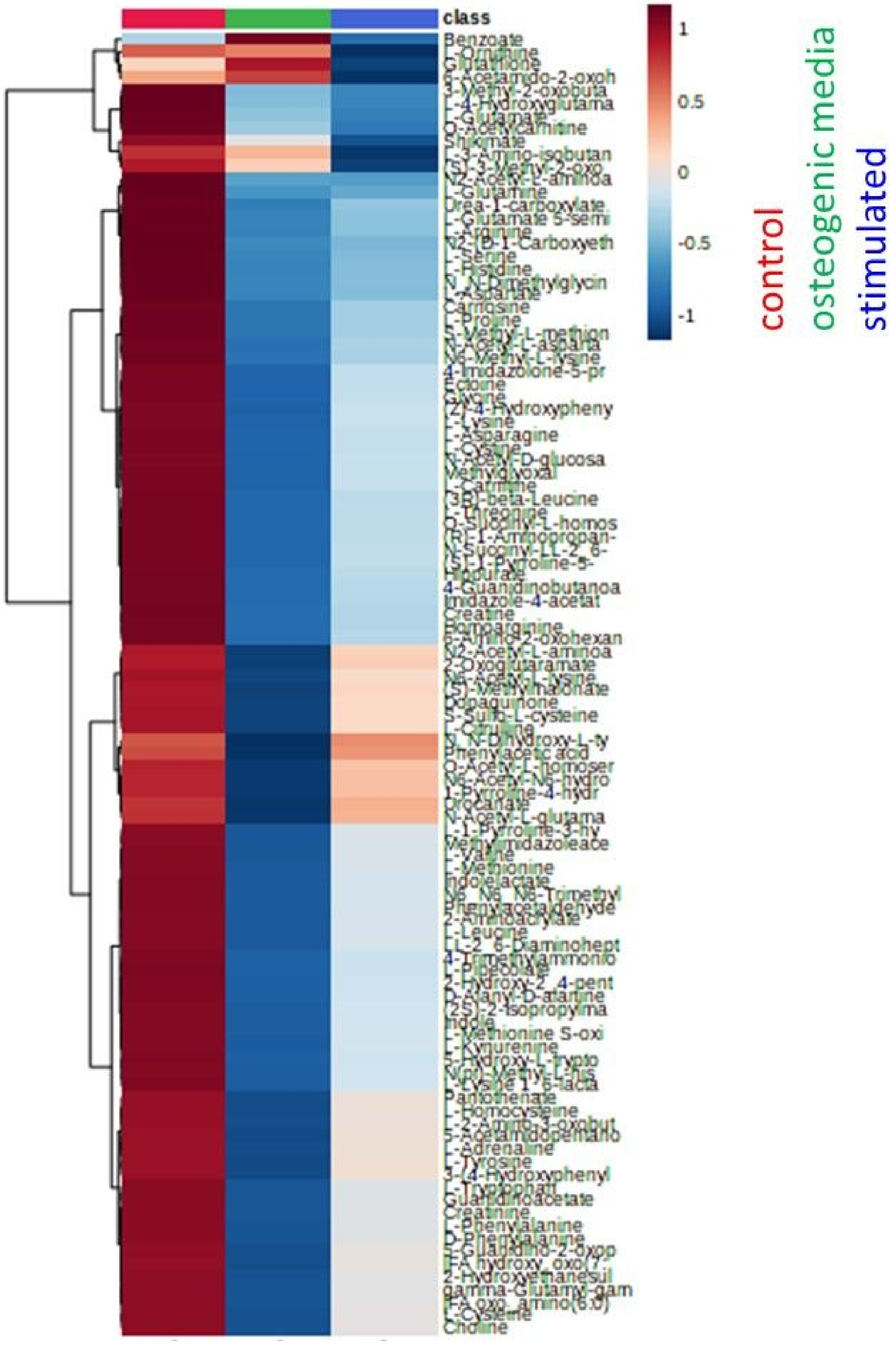

Figure S4. Amino acids heatmaps of metabolomic analysis of MSCs cultured on non-stimulated control (non-stimulated on PVDF-CFO), osteogenic control (osteogenic media on glass slides) and with stimulation on PVDF-CFO in balanced media for 7 days.

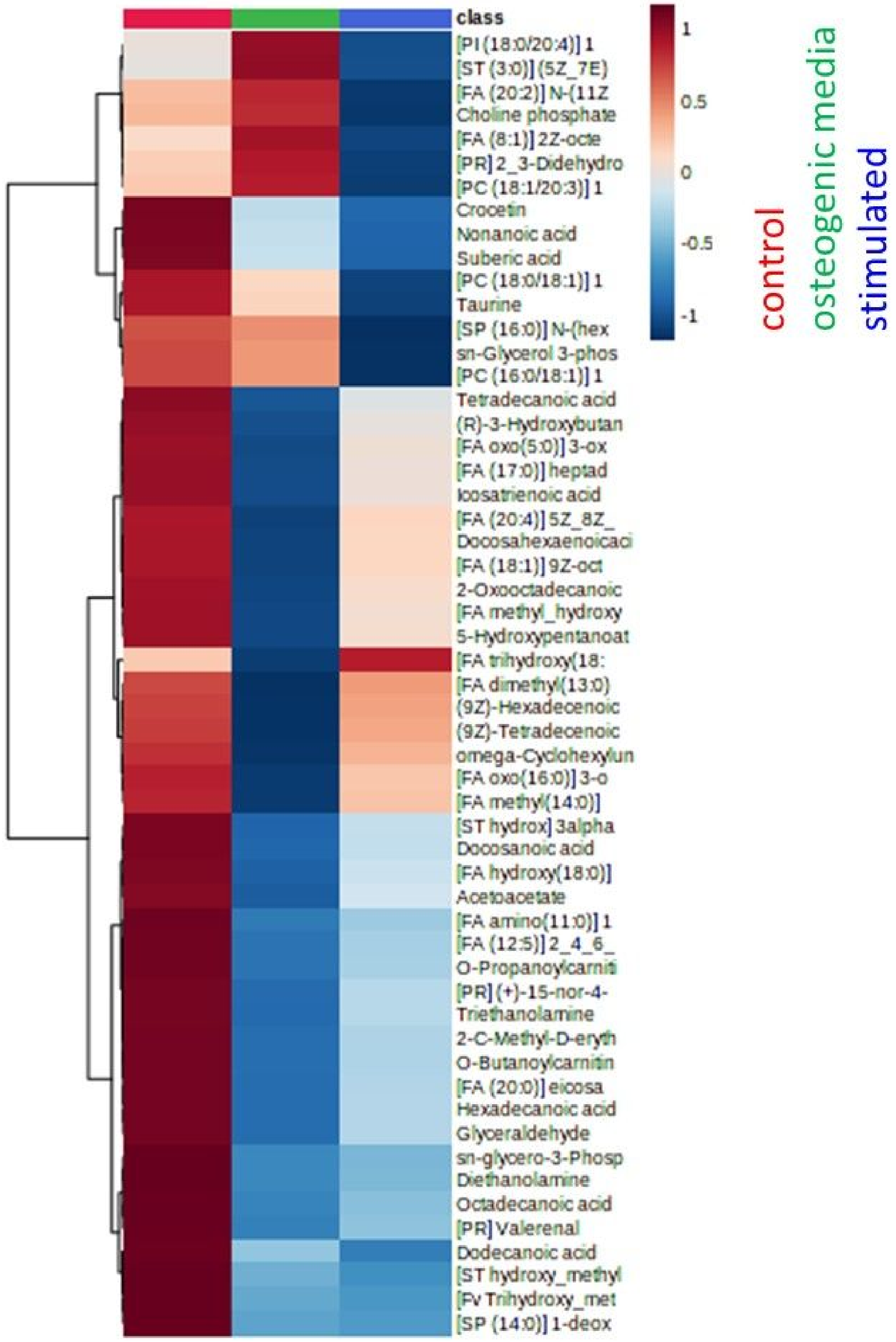

Figure S5. Lipids heatmaps of metabolomic analysis of MSCs cultured on non-stimulated control (non-stimulated on PVDF-CFO), osteogenic control (osteogenic media on glass slides) and with stimulation on PVDF-CFO in balanced media for 7 days.

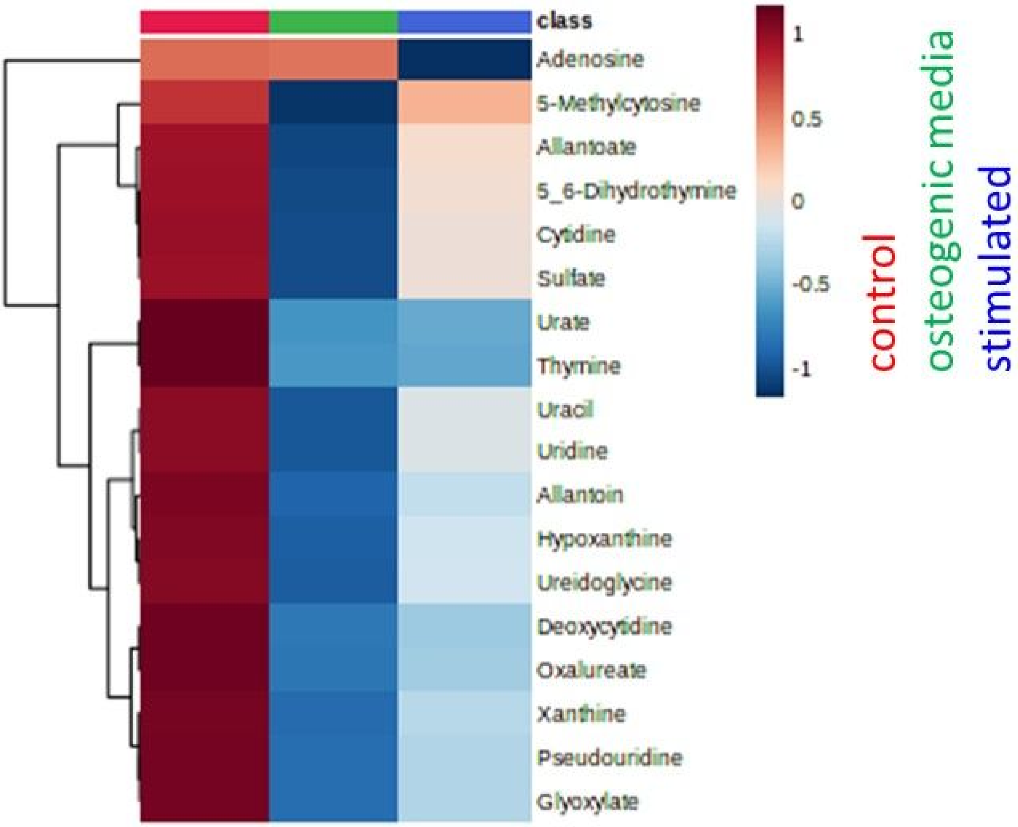

Figure S6. Nucleotides heatmaps of metabolomic analysis of MSCs cultured on non-stimulated control (non-stimulated on PVDF-CFO), osteogenic control (osteogenic media on glass slides) and with stimulation on PVDF-CFO in balanced media for 7 days.

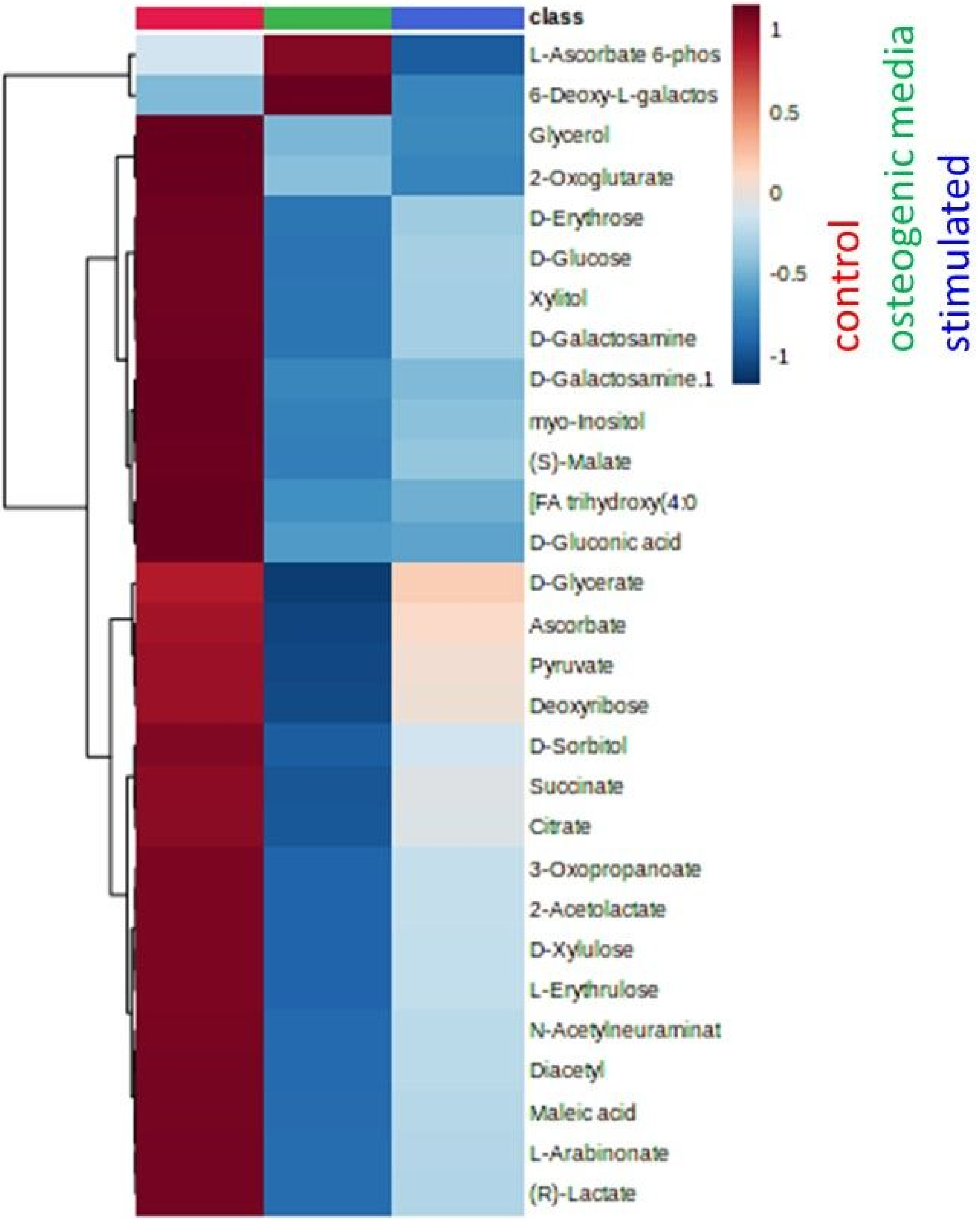

Figure S7. Carbohydrates heatmaps of metabolomic analysis of MSCs cultured on non-stimulated control (non-stimulated on PVDF-CFO), osteogenic control (osteogenic media on glass slides) and with stimulation on PVDF-CFO in balanced media for 7 days.

REFERENCES

(1) Kilian, K. A.; Bugarija, B.; Lahn, B. T.; Mrksich, M. Geometric Cues for Directing the Differentiation of Mesenchymal Stem Cells. *Proc. Natl. Acad. Sci.* **2010**, *107* (11),

4872–4877. https://doi.org/10.1073/pnas.0903269107.

(2) Einhorn, T. A.; Gerstenfeld, L. C. Fracture Healing: Mechanisms and Interventions. *Nat. Rev. Rheumatol.* **2015**, *11* (1), 45–54. https://doi.org/10.1038/nrrheum.2014.164.

(3) Gómez-Barrena, E.; Rosset, P.; Lozano, D.; Stanovici, J.; Ermthaller, C.; Gerbhard, F. Bone Fracture Healing: Cell Therapy in Delayed Unions and Nonunions. *Bone* **2015**, *70*, 93–101. https://doi.org/10.1016/j.bone.2014.07.033.

(4) McBeath, R.; Pirone, D. M.; Nelson, C. M.; Bhadriraju, K.; Chen, C. S. Cell Shape, Cytoskeletal Tension, and RhoA Regulate Stem Cell Lineage Commitment. *Dev. Cell* **2004**, *6* (4), 483–495. https://doi.org/10.1016/S1534-5807(04)00075-9.

(5) Yang, Y.; Wang, X.; Wang, Y.; Hu, X.; Kawazoe, N.; Yang, Y.; Chen, G. Influence of Cell Spreading Area on the Osteogenic Commitment and Phenotype Maintenance of Mesenchymal Stem Cells. *Sci. Rep.* **2019**, *9* (1), 6891. https://doi.org/10.1038/s41598-019-43362-9.

(6) Biggs, M. J. P.; Richards, R. G.; Gadegaard, N.; Wilkinson, C. D. W.; Dalby, M. J. Regulation of Implant Surface Cell Adhesion: Characterization and Quantification of S-phase Primary Osteoblast Adhesions on Biomimetic Nanoscale Substrates. *J. Orthop. Res.* **2007**, *25* (2), 273–282. https://doi.org/10.1002/jor.20319.
